# Resolved frustrated tunneling ionization (FTI) in asymmetrical fast oscillation of above-threshold ionization spectrum

**DOI:** 10.1016/j.isci.2025.111899

**Published:** 2025-01-25

**Authors:** Lifeng Wang, Hao Teng, Fei Li, Bingbing Wang, Xiaoxin Zhou, Peng He, Zhiyi Wei

**Affiliations:** 1 Xi’an Institute of Applied Optics, Xi’an, China; 2 Beijing National Laboratory for Condensed Matter Physics, Institute of Physics, Chinese Academy of Sciences, Beijing, China; 3 Institute of Advanced Science Facilities, Shenzhen, China; 4 Songshan Lake Materials Laboratory, Dongguan, China; 5 Research Center for Advanced Optics and Photoelectronics, Department of Physics, College of Science, Shantou University, Shantou, Guangdong, China; 6 University of Chinese Academy of Sciences, Beijing, China; 7 College of Physics and Electronic Engineering, Northwest Normal University, Lanzhou 730070, China

**Keywords:** Physics, Optics, Laser

## Abstract

We experimentally investigated the carrier-envelope phase (CEP) effect on above-threshold ionization (ATI) process of argon in frustrated tunneling ionization regime. For the first time, we found the asymmetrical fast oscillation of the ionization probability with a period around π/6 in ATI spectrum, to the best of our knowledge. Simulation results agreed well with the experimental data. Two kinds of electron trajectories were resolved to interpret the experimental observations. One was the tunneling ionization directly from the ground state, which was responsible for the asymmetry in spectrum, while the other one was tunneling ionization from the excited states, which contributed to the π/6 oscillation. Our results served as evidence that the bound states population was controlled by the CEP.

## Highlights

• Asymmetrical ATI spectrum from argon in terms of CEP is experimentally observed
• Fast oscillation with a period around π/6 in the ATI spectrum is demonstrated
• A semi-classical interpretation is proposed in frustrated tunneling ionization regime

## Graphical abstract

**Figure:**
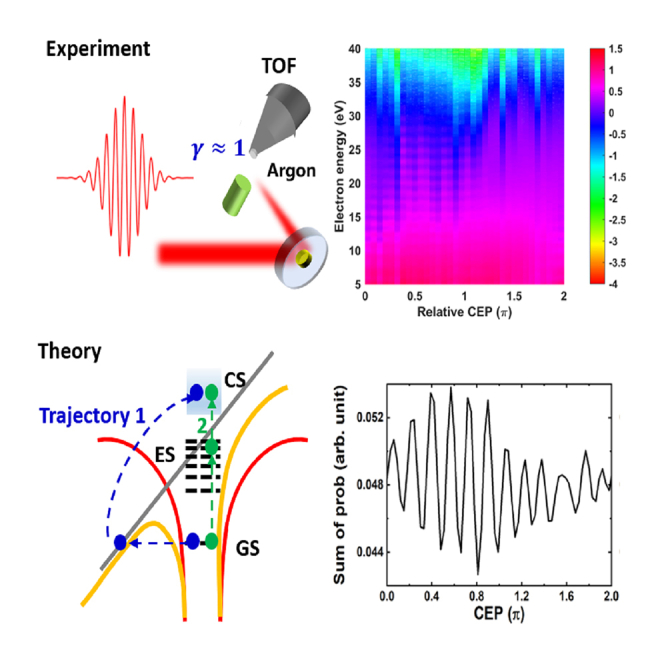

## Introduction

When atoms or molecules are exposed to intense ultrafast lasers, the tunneling ionization of electron is a fundamental process for several physical phenomena, such as above-threshold ionization (ATI), filamentation, and high-order harmonic generation (HHG).^1^^,^^2^^,^^3^ Commonly, the tunneling rate is equal to ionization rate. However, this is not true if the Coulomb potential of the parent ion is considered.^4^^,^^5^ Attracted by the Coulomb potential, the electron has possibility to have a total negative energy at the tunnel exit and relaxes to bound states; this process is dubbed as frustrated tunneling ionization.^6^^,^^7^^,^^8^^,^^9^^,^^10^ The bound states of atoms or molecules have been proved to play important roles in many different dynamic processes in intense laser fields, such as acceleration of neutral atoms,^11^ measurement of laser intensity,^12^ manipulation of quantum states of mesoscopic size,^13^ below threshold HHG,^14^ etc. There are two mechanisms of the formation of excited states in atoms or molecules, which are still under debate.^15^^,^^16^^,^^17^^,^^18^^,^^19^^,^^20^ One is that excited states are populated by multiphoton transition,^21^ while the other one considers that the electron is recaptured into excited states after tunneling ionization.^22^ From the experimental point of view, the formation of excited states is usually investigated in terms of laser intensity with multicycle femtosecond laser pulses.^23^^,^^24^

For ultrashort laser pulses, especially in few-cycle regime, there is an important parameter CEP, proved to be critical in many physical phenomena.^25^^,^^26^^,^^27^ However, the CEP effect on the formation of excited states in atoms or molecules has not been extensively investigated so far. T. Nakajima et al.^28^ theoretically investigated the CEP dependent bound state atoms in the multiphoton ionization regime, but without experimental evidence. H. Yun et al.^29^ observed HHG from hydrogen by 5-fs laser pulses. The authors contributed the coherent extreme-ultraviolet emission to the excited atoms through the frustrated tunneling ionization, where the population of bound states was calculated in terms of CEP. J. Liu et al.^30^ theoretically optimized the CEP for the creation of bound states. Recently, M Kübel et al.^31^ experimentally observed that the modulation periodicity of ATI spectrum from Cs in gas-phase driven by a 3.1-μm laser is 2π/3. The fast oscillation happened in the energy range of [5Up, 7Up] in the experiment, where Up was the ponderomotive energy. The authors explained that the unusual behavior originated from the interference of few recollision quantum orbits of the ionized electron, which was independent with Rydberg states excitation process. D. Chetty et al.^32^ experimentally measured the excited argon atoms (Ar∗) generated by few-cycle laser pulses and observed a different behavior of bound-state population in terms of CEP at low and high laser intensities. The boundary found in experiment is caused by the transition from the multiphoton to the tunneling regime. Their results served as a proof of principle that bound-state populations were controlled by precisely engineered pulses.

Here we reported experimental data of asymmetrical fast oscillation of ATI spectrum from argon in terms of CEP. We observed a modulation of ATI photoelectron spectrum with a period around π/6 for relative CEP in range of 0 to π, which was the fastest CEP-induced modulation period, to the best of our knowledge. Interestingly, this modulation decreased dramatically in the range of π to 2π. Our simulation results, which were well agreed with experimental data, resolved the contributions of two different electron trajectories in the frustrated tunneling ionization regime. One was the electron tunneling ionized directly from the ground state in the asymmetrical laser field, which was responsible for the asymmetry in spectrum. The other one was the electron tunneling ionized from the excited states. Our simulation results showed that there was a phase shift between the neighboring excited states and the fast oscillation came from the interferences of electron in all excited states. Our results served as evidence that the excited states population was controlled by the CEP. Meanwhile, the asymmetrical dependence of the ATI spectrum on the CEP may provide a potential way to measure the absolute CEP with only one electron time-of-flight (TOF) spectrometer.

## Results and discussion

### Experimental setup and methods

Our experiment was performed on a system of isolated attosecond pulse measurement in Institute of Physics, Chinese Academy of Sciences.^33^ A commercial Ti: sapphire chirped-pulse amplifier (Femtolaser Compact PRO) provided laser pulses with central wavelength of 760 nm, pulse duration of 25 fs, pulse energy of 0.8 mJ laser pulses at 1 kHz repetition rate. The laser spectrum was broadened in a differentially pumped neon-filled hollow-core fiber and then temporally compressed by chirped mirrors.^34^ The whole spectrum range covered from 550 nm to 950 nm. The CEP of the laser pulse was actively stabilized with a fluctuation of 85 mrad in more than 7 h by locking both the oscillator and the amplifier loops.^35^ Finally, laser pulses with energy of 0.25 mJ and duration around 7 fs FWHM were obtained.^26^ The stability of our system ensured the successful measurements of isolated attosecond pulse^36^ and free electron density in filamentation driven by few-cycle laser pulses.^26^

The linearly polarized laser pulses were focused into a vacuum chamber with backing vacuum pressure of 10^−7^ mbar. A motorized aperture was set to control the laser power, which was important in our experiment. The infrared (IR) laser was focused by a two-segment-mirror, consisted by an inner Mo/Si mirror and an outer silver mirror. The delay between the Mo/Si mirror and silver mirror was fixed in our experiment around zero delay. The pulse duration was measured around 7 fs before the two-segment mirror. The minimum step of the delay was 5 ± 1 nm, corresponding to 16.7 ± 3.3 as, meaning that the pulse duration was not significantly stretched by the mirror. Therefore, the total IR laser field was synthesized by the two IR laser beams, and this configuration gave freedom to modify the asymmetry of total IR laser field. As discussed in the following, the asymmetrical laser field played a key role in our experimental observations, and this was a major difference from similar experimental setups.^32^^,^^37^^,^^38^^,^^39^ The IR intensity at focus was controlled by the motorized aperture from 10^11^ W/cm^2^ to 10^14^ W/cm^2^. In the range of focus, Argon atoms were injected by a gas jet to interact with IR laser to trigger ATI process. The photoelectron spectrum for ATI was recorded with a TOF spectrometer in terms of the CEP of the few-cycle IR laser (see supplemental material for more details).

**Figure 1:**
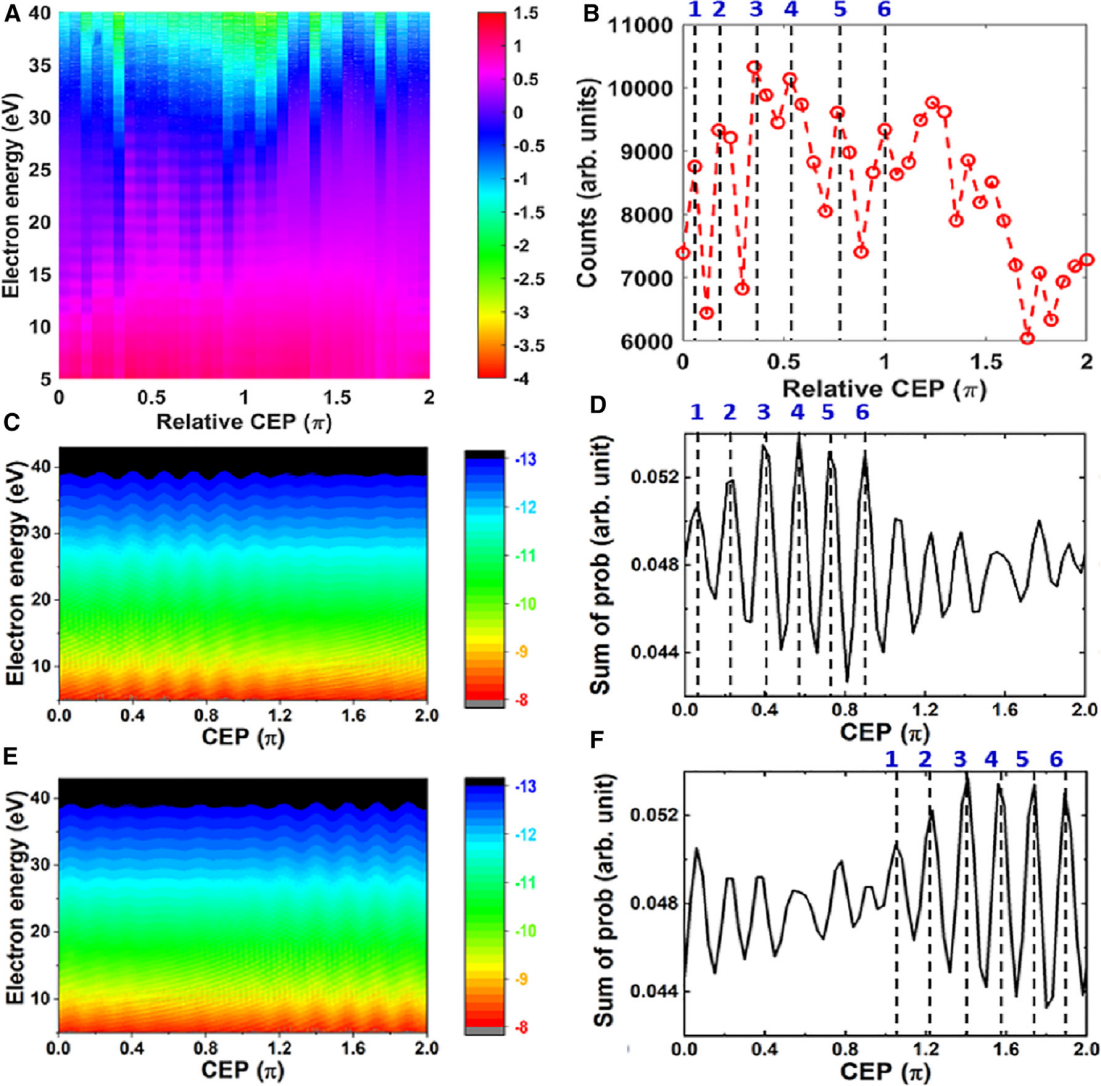
Experimental and theoretical ATI spectra of argon (A and B) The measured ATI spectra of argon (A) and total ATI counts in range of 5–40 eV (B) as a function of relative CEP driven by few-cycle laser pulses. (C–F) Calculated ATI spectra vs. CEP along the positive (C) and negative (E) directions of laser’s polarization. Summation of ionization probability vs. CEP along the positive (D) and negative (F) directions of laser’s polarization.

The measured ATI spectra in logarithm scale as a function of CEP were shown in Figure 1A, in which CEP was tuned with a step of π/17, which was calibrated by HHG spectrum.^40^ The measurement range of the TOF was from 5 to 200 eV. The data acquisition time was 2 s, meaning one ATI spectrum was averaged by 2,000 laser pulses at a given CEP. The total ATI counts in energy of 5–40 eV in terms of CEP was shown in Figure 1B. There were several interesting observations in the experimental results. Firstly, oscillation in ATI spectrum was found with a period around π/6 in the range of 0 to π, as marked by dashed lines in Figure 1B. The Nyquist frequency of the CEP scan was 8.5/π, which was higher than the frequency measured. Secondly, another striking feature of our experimental results was that the oscillation decreases dramatically in the range of π to 2π. We have observed similar phenomena with IR and extreme ultraviolet (XUV) pulses (see supplemental material for more details). Compared to experimental results in a study by Kübel et al.,^31^ the CEP-dependent oscillation repeated with a period of 2π/3 in a range of 0 to 4π. The differences between the observations from the study by Kübel et al.^31^ and our experiment indicated that there were different underlying mechanisms.

**Figure 2:**
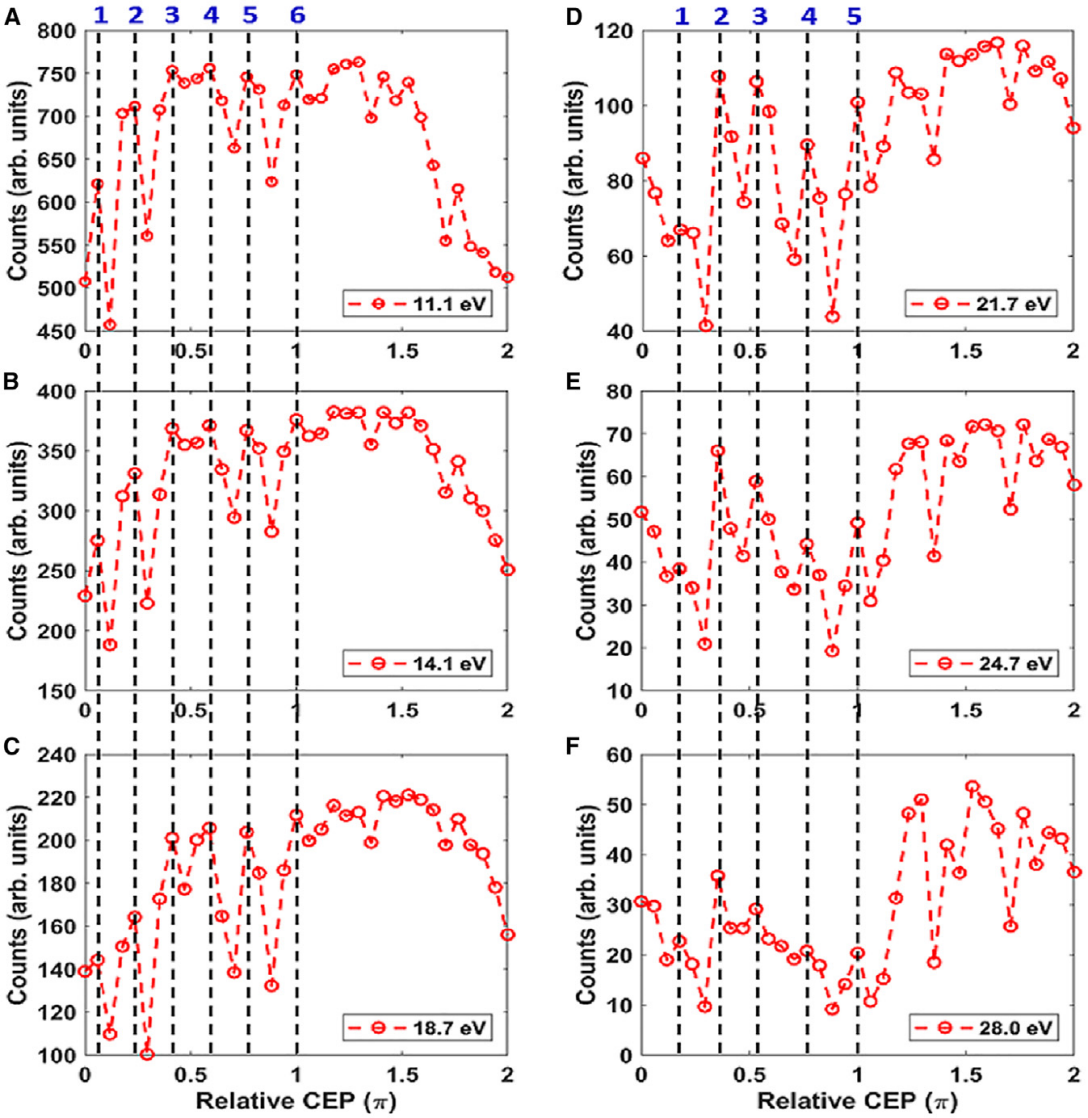
The measured ATI spectrum of Argon as a function of CEP at different electron energy range Experimental ATI spectra vs. CEP in electron range of 11.1 (A), 14.1 (B), 18.7 (C), 21.7 (D), 24.7 (E), and 28 eV (F).

To see our experimental finding more clearly, the measured ATI electron counts at different energy ranges (±0.8 eV) were shown in Figure 2 (see supplemental material for other energy ranges). For electron energies at 11.1, 14.1, and 18.7 eV, the ATI counts oscillated six times in the range of 0 to π, as shown in Figures 2A–2C. While for electron with energies at 21.7, 24.7, and 28 eV, there are five oscillations in the range of 0 to π, as shown in Figures 2D–2F. However, for all energies selected in Figure 2, the oscillation of ATI counts as a function of CEP became less significant in range of π to 2π. This deviation may come from experimental error since there are more ATI counts at lower energy range than that at higher energy range. As a result, the total ATI counts evolved six times of oscillation in the range of 0 to π, as shown in Figure 1B. The origination for experimental observations will be discussed in the following.

To understand the experimental results, one-dimensional time-dependent Schrödinger equation (TDSE) was solved to describe the interaction between the atom and the linearly polarized laser pulses. The electric field of the laser pulses was expressed as

(Equation 1) $$E\left(t\right)=\sum_{i}\;E_{i}f\left(t\right)\sin\left(\omega_{i}t+\phi \right)$$

where $E_{i}$ was the electric field amplitude, $f\left(t\right)=\sin^{2}\left(\pi t/\tau \right)$ was the electric field envelope with τ being the pulse duration of the laser pulse, $\omega_{i}$ was the frequency of the laser pulse, and ϕ was the CEP of the laser pulse. The initial state was obtained by diagonalizing the field-free Hamiltonian $H_{0}$ of the atom and the TDSE was solved by the split-operator method. Hence, the population of the bound states after the end of laser pulse was obtained by projecting the final wave function to the eigenstates of $H_{0}$. We would like to emphasis that the bound states connect the excitation and ionization of a single electron within the laser pulses. As we explain in the following, the bound states are critical to interpret the experimental observations.

### Experimental and theoretical results

Our simulations clearly reproduced the main characterizations in experimental results, as shown in Figures 1C–1F. In experiment, we measured the pulse duration around 7 fs while the whole spectrum covered from 550 to 950 nm. To constructed laser field, three-color laser field for wavelength of 660 nm, 760 nm, and 860 nm with same CEP and pulse duration was used and the intensities at 660 nm and 860 nm were 36% of that at 760 nm. The other calculation parameters were 7.8 fs (FWHM) and peak intensity of $1.9\times 10^{14}\;W/{\text{cm}}^{2}$ for 760 nm. The simulation results of ATI spectra generated by 5 fs will also be discussed in the following. We should mention that oscillation structure in the spectra of Figures 1C and 1E was still observable with including the influence of focal volume averaging^24^^,^^39^^,^^41^ in our simulation, where the intensity region was from $0.1\times 10^{14}\;W/{\text{cm}}^{2}$ to $2.0\times 10^{14}\;W/{\text{cm}}^{2}$ with the intensity varied in step of $5.0\times 10^{12}\;W/{\text{cm}}^{2}$ (see supplemental material for calculation results). Because there was one TOF spectrometer in our experiment along laser’s polarization direction, only electrons along one direction were summed in the calculation. The ATI spectrum exhibited oscillations with a periodicity of π/6 within the range of 0 to π, and this oscillation notably diminished within the range of π to 2π, as illustrated in Figure 1C. Furthermore, the ATI electron counts along another direction as a function of CEP were shown in Figure 1E, which was not measured in experiment, the oscillation appeared in the range of π to 2π. To see this oscillation more clearly, the total ionization probability along two directions in terms of CEP were shown in Figures 1D and 1F with the energy of the electron in region of 5–40 eV, marked by dashed lines in Figures 1B and 1D. The qualitatively agreement between the simulation results and experimental data give rise to a question: what is the underlying mechanism of these experimental observations?

**Figure 3:**
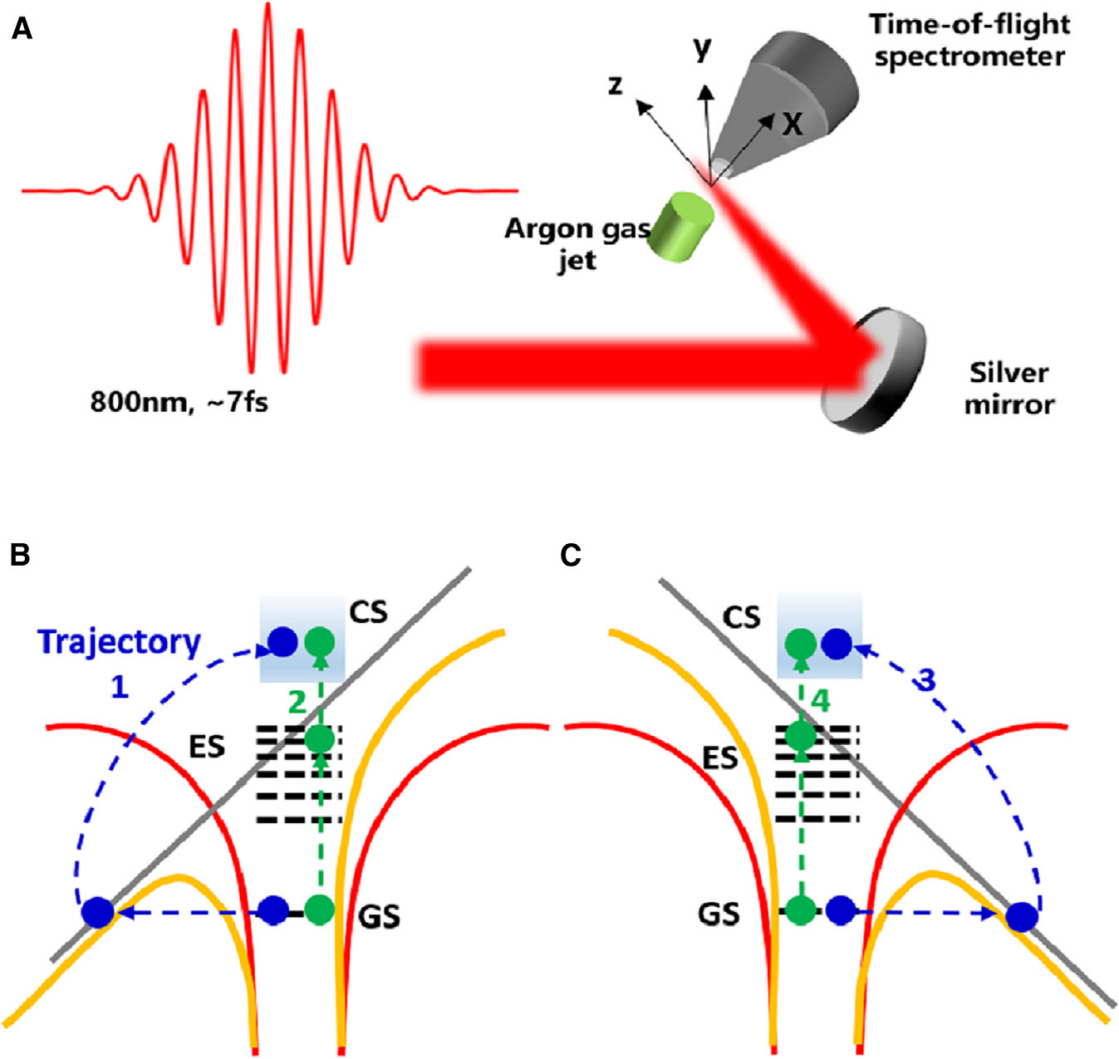
Demonstration of two electron trajectories in ATI spectrum in frustrated tunneling ionization regime (A) The schematic of simplified experimental setup. (B) Two trajectories of electrons ionization along +x direction. (C) Two trajectories of electrons ionization along −x direction. GS, ground state, CS, continuum state; ES, excited state. The red, gray and yellow lines are the Coulomb potential, the external laser field and the total electric field, respectively in (B) and (C).

Here, we proposed a semi-classical picture of electron ionization, as illustrated in Figure 3. We simplified the experimental setup, as shown in Figure 3A. The ionization of electron was discussed under two conditions. On one hand, when the electric field oscillated along +x direction, as shown in Figure 3B, the electron was tunneling ionized from ground state directly, which was labeled as trajectory 1. On the other hand, the electron had possibility to be populated by the combined effect of laser electric field and Coulomb potential into excited states rather than tunneling. Later, the electron may be further ionized from excited states to continue state by laser field, which was labeled as the trajectory 2. The ionized electrons from both trajectories 1 and 2 were measured in experiment. When the electric field oscillated along −x direction, as shown in Figure 3C, there were another two trajectories, marked as trajectory 3 and 4, similar to Figure 3B. For trajectories 1 and 3, the electrons moved along +x and −x directions, which depended on the electric field. However, since the Coulomb potential was spatially symmetry, both trajectories 2 and 4 may contribute to a certain excited state, and the ionization from excited state was independent with the direction of laser’s polarization because it was multiphoton ionization process from these excited states. These characteristics of the trajectories play important roles in our experimental results, as discussed in the following.

**Figure 4:**
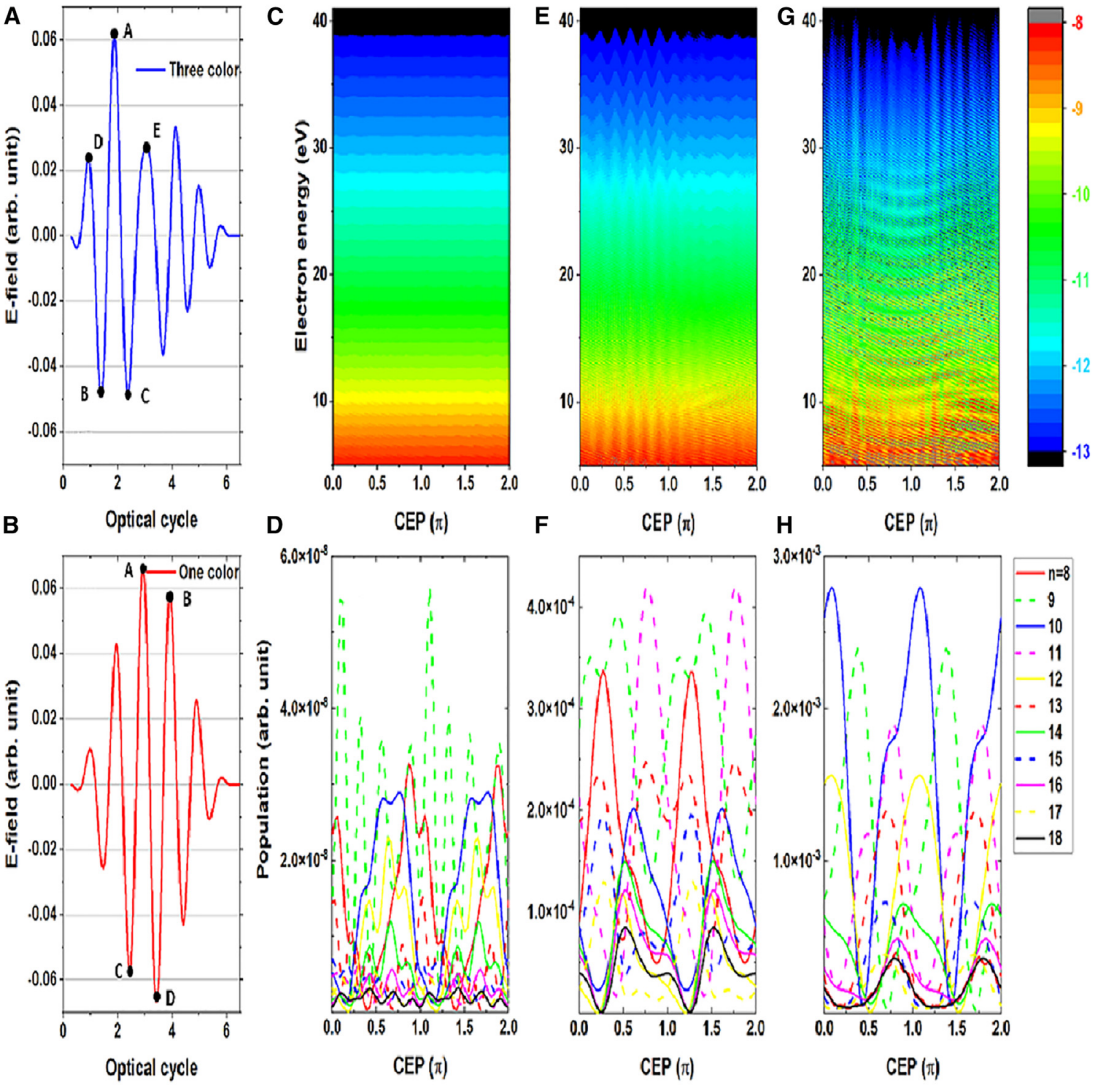
Simulation results of ATI spectrum driven by 7.8 fs laser pulses (A and B) Electric filed with three-color (A) and one-color (B) driving laser pulse. (C–F) Calculated ATI spectrum and corresponding population of bound states with three-color driving laser field at intensity of 4 × 10^13^ W/cm^2^ (C and D), and intensity of 1.9 × 10^14^ W/cm^2^ (E and F). (G and H) Calculated ATI spectrum and corresponding population of bound states with one-color driving laser field at intensity of 1.4 × 10^14^ W/cm^2^.

To explore the underling mechanism of the one-dimensional TDSE results, ATI spectra under different conditions were calculated. Firstly, we investigated the laser intensity effect on the ATI spectrum driven by three-color (660 nm, 760 nm, and 860 nm with relative amplitude of electric field 0.6:1:0.6) laser field, as shown in the Figure 4A. To understand the oscillation period, the population of atoms in excited states was also calculated. When the laser intensity was 4 × 10^13^ W/cm^2^, the electrons were mostly ionized by multiphoton ionization. In multiphoton ionization regime, the ionization probability was independent to the population of the excited states. Therefore, although the population of excited states with the principle number of odd and even oscillated with CEP, as shown in Figure 4D, we cannot observe any oscillation in the ATI spectrum, as shown in Figure 4C. However, when the laser intensity was increased to 1.9 × 10^14^ W/cm^2^, the tunneling ionization dominated the electron ionization process. Electrons from both trajectories 1 and 2 started to play an important role. The populations of excited states increased four orders of magnitude (∼10^−4^), as shown in Figure 4F. More importantly, the CEP dependent on odd symmetry and neighboring even symmetry excited states had a phase shift around π/4. By using different absorbing boundaries in calculation, one may find that the contribution mainly came from excited states with principle number larger than 7 (see supplemental material for more details). Although the populations of the excited states exist π/4 dephasing, the situation becomes more complex that cannot predict only by this dephasing, the reason is as follows: on one hand, from Figure 4F we may find that there are two peaks for some states as a function of CEP in the region of 0 to π; on the other hand, the parity effect of the excited states started to break down under the laser conditions in our experiment, which was also demonstrated in studies by Chetty et al.^32^ and Venzke et al.^42^ Consequently, the total ionization probability had an oscillation of π/6 by the interference coming from the electrons of trajectories 1 and 2, as shown in Figure 4E.

Secondly, we performed simulation with one-color (760 nm) laser field, as shown in the Figure 4B to compare with that generated by three-color laser field. The oscillation of ATI spectrum with CEP was clearly observed for one-color laser field at intensity of 1.4 × 10^14^ W/cm^2^, as shown in Figure 4G, where there was no different between CEP∈[0, π] and CEP∈[π, 2π]. The calculation results could be understood by the symmetry of electric field. For one-color laser field, there were more than one peaks to contribute the population of trajectory 1, like A and B in positive direction or C and D in negative direction, as shown in Figure 4B. The interference between electrons of trajectory 1 in different optical cycles washed away the asymmetrical dependence of ATI spectrum on CEP. However, for the three-color laser field, there was only one dominate peak A in positive direction and two peaks B and C in negative direction, as shown in Figure 4A. The amplitude of A was twice the amplitudes of D and E in the same direction; therefore, the three-color laser field was asymmetrical not only in terms of the CEP but also in the behavior of the electrons following trajectory 1. The comparison of ATI spectrum driven by one-color and three-color lasers gave a clear picture on the contributions of electrons from two different trajectories. The interference of electrons from trajectory 2 caused the CEP dependent oscillation in ATI spectrum, while the electrons from trajectory 1 break the symmetry of the ionization spectra for relative CEP∈[0, π] and [π, 2π] because of the asymmetrical electric field.

**Figure 5:**
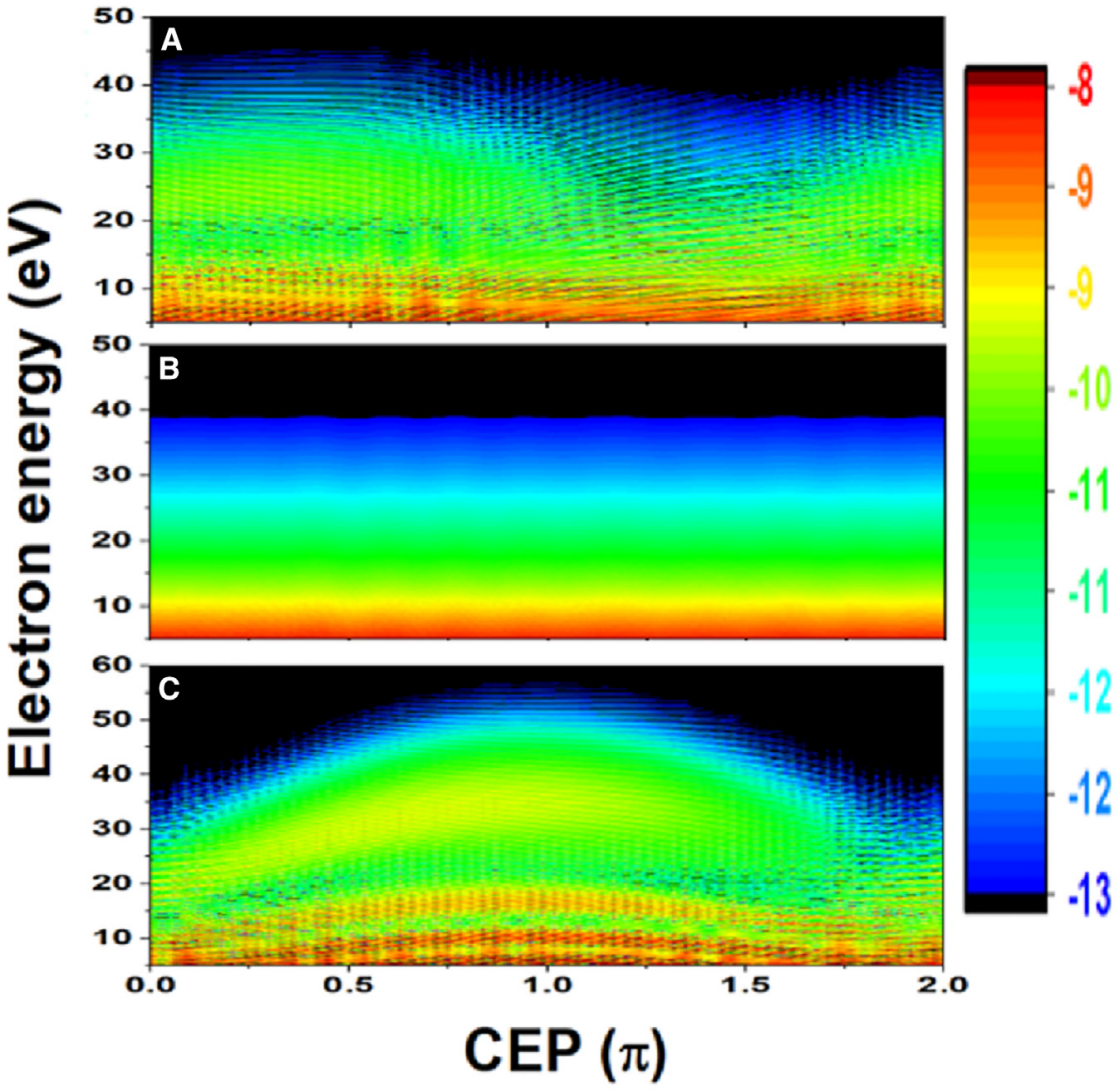
Simulation results of ATI spectrum under different conditions Calculated ATI spectrum driven by three-color lasers with 3 optical cycles at intensity of 3.8 × 10^14^ W/cm^2^ (A); by three-color laser with 2 optical cycles at intensity of 4 × 10^13^ W/cm^2^ (B); by three-color laser with 2 optical cycles at intensity of 1.9 × 10^14^ W/cm^2^ (C).

Lastly, to verify our understanding, the laser intensity was experimentally increased to 3.8 × 10^14^ W/cm^2^ and it was found that the fast CEP-dependent oscillation disappears (see supplemental material for more details). The corresponding calculated ATI spectrum was shown in the Figure 5A. It was found that there was no oscillation in ATI spectrum, where the trajectory 1 dominated and the trajectory 2 could not significantly affect the ionization probability at this intensity. Furthermore, we theoretically investigated the effect of pulse duration to the ATI spectrum. When the pulse duration decreased from 7.8 fs (three optical cycles) to 5.2 fs (two optical cycles), the ATI spectrum changed quite a lot. At intensity of 4 × 10^13^ W/cm^2^, the ATI spectrum was independent of CEP, as shown in Figure 5B, which was similar to that of the Figure 4C. As the intensity increased to 1.9 × 10^14^ W/cm^2^, there was no CEP-dependent modulation but a totally different structure in ATI spectrum, as shown in Figure 5C. Other effects including radial chirp of driven laser after hollow core fiber and Gouy-phase flip may induce complex spatiotemporal structure at the focus of the two-segment mirror. These effects were negligible under our experimental conditions. Efforts are payed to perform three-dimensional simulation to make the simulation results more convincing (see supplemental materials for calculation results). Our simulation results indicated that our experimental results were quite sensitive to the laser parameters, and fruitful physical phenomena may happen under different experimental conditions.

It is interesting to compare our results to the work of Chetty et al.^32^ In our experiment, the ionized electrons of argon generated by few-cycle laser pulses were measured but the excited argon (Ar∗) atoms were not measured. The laser pulse duration was ∼7.8 fs (FWHM) and had asymmetrical structure (as shown in Figure 4A) in our work, while the pulse duration was ∼6 fs in the study by Chetty et al.^32^ Difference electric fields resulted to the different experimental oscillation structures, π/6 in our work and π in the study by Chetty et al.^32^ Theoretically, our calculation results based on one-dimensional TDSE showed that the parity effect breaks down for principle quantum number at laser intensity of 1.9 × 10^14^ W/cm^2^, which was also observed in Chetty’s work. Together, these two works presented evidence that the bound state populations were controlled by CEP in few-cycle regime. Additionally, compared to the simple-man model, which gave intuitive physical picture and analytic approximation to explain ATI, HHG, non-sequential multielectron ionization and so on,^43^^,^^44^^,^^45^^,^^46^^,^^47^ there were two differences in our simulation. Firstly, soft-core Coulomb potential was used and secondly bound states were considered, which were critical to explain the experimental results.

Last but not least, our experimental findings may provide a potential method to measure the absolute CEP with only one TOF, in contrast to the conventional and enhanced “stereo-ATI” experiments^48^^,^^49^ that typically require more complex setups. Calibrated by the numerical results, the absolute CEP of few-cycle laser pulse can be determined because of the asymmetry in the measured ATI spectrum. In a study by Khurmi et al.,^50^ they also proposed a method to measure the absolute CEP with one TOF. Their method relied on that the Schrödinger equation of hydrogen atom in the strong laser field can be numerically solved with high precision. By comparing the experimental ATI spectrum from hydrogen atom with the simulation results, the absolute CEP was determined. However, with other noble gases, significant differences were observed between experimental data and simulation results. Compared with the study by Khurmi et al.,^50^ our experimental data were obtained in argon atoms, which may be provide a more versatile way to measure the absolute CEP. However, precise experimental parameters, including laser pulse duration, intensity, beam profile and sophisticated 3D simulation are needed to improve the accuracy.

## Discussion

In summary, ATI spectrum from argon driven by few-cycle IR laser pulses was experimentally investigated when the Keldysh parameter was close to 1. For the first time, we observed a π/6 oscillation of whole ATI spectrum for relative CEP∈[0 π], to the best of our knowledge. Interestingly, this oscillation dramatically decreased for range of π to 2π under our experimental conditions. The simulation results were well agreed with our experimental data qualitatively. We proposed a semi-classical physical picture and contributed the experimental results to two kinds of electrons with different trajectories. The interference of electrons ionized from excited states caused the fast oscillation while the electrons ionized directly by tunneling from the ground state were responsible for the asymmetry in ATI spectrum. Our results helped to understand the physical mechanism of CEP modulated FTI process in strong laser field. Our proposed physical picture in FTI regime is one possible explanation to experimental results, more sophisticated simulation results are needed to get deeper understanding mechanism.

### Limitations of the study

In our experiment, we use only one TOF spectrometer to measure ionized electrons’ kinetic energy along laser’s one polarization direction. The information of electrons’ kinetic energy along another polarization direction is missing. In future, we plan to collect this missing data to verify and deeply modify our proposed mechanism.

## Resource availability

### Lead contact

Further information and requests for resources and reagents should be directed to and will be fulfilled by the lead contact, Bingbing Wang (wbb@iphy.ac.cn).

### Materials availability

This work did not generate new materials.

### Data and code availability

• All data reported in this paper will be shared by the lead contact upon request.
• This paper does not request original code.
• Any additional information required to reanalyze the data reported in this paper is available from the lead contact upon request.

## Acknowledgments

The authors thank Zenghu Chang for useful suggestions and Weichao Jiang for three-dimensional TDSE calculation results. B.W. thanks Jing Chen for fruitful discussion. The work is financially supported by the 10.13039/501100001809National Natural Science Foundation of China (NSFC Grant Nos. 12034020, 12074418, 11774411, 11864037, 12204526, 91850209, 92250303, 12474288, and 62475172), STU Scientific Research Initiation Grant (No. NTF23011), and 10.13039/501100012166National Key R&D Program of China (Grant No. 2022YFA1604200).

## Author contributions

Conceptualization, Z.W., H.T., and L.W.; methodology, Z.W. and H.T.; investigation, L.W., F.L., B.W., and P.H.; writing – original draft, L.W., B.W., and F.L.; writing – review & editing, H.T., X.Z., P.H., and Z.W.; funding acquisition, H.T., B.W., and Z.W.; resources, H.T., B.W., and Z.W.; supervision, H.T., B.W., and Z.W.

## Declaration of interests

The authors declare no competing interests.

## STAR★Methods

### Key resources table

**Table:** 

REAGENT or RESOURCE	SOURCE	IDENTIFIER
**Equipment in experiment**		

CPA Laser	Femtolasers Produktions GmbH	Femtopower compact PRO
Time of flight spectrometer	Stefan Kaesdorf	ETF 10
Two-segment-mirror	Ultrafast innovation	NA

### Method details

#### Laser source

The laser source is a near-infrared chirped-pulse amplifier, which delivers laser pulses with central wavelength of 760 nm, pulse energy of 0.8 mJ, pulse duration of 25 fs at a repetition rate of 1 kHz.

#### Spectrum broadening

Near-infrared femtosecond laser pulses is focused into a 1-m hollow core fiber to interact with neon. Neon is injected into the hollow core fiber at a pressure of 2.5 bar at one end and differentially pumped out at the other end where laser enters. The broadened spectrum covers from 550 nm to 950 nm.

### Quantification and statistical analysis

#### Detection of kinetic energy of electrons

The kinetic energy of electrons is detected by a TOF spectrometer (model: ETF 10, company: Stefan Kaesdorf). The measured range of the TOF spectrometer is 5–200 eV, which covers the kinetic energy of electrons in our experiment. At a fixed CEP, we measured the kinetic energy of electrons within a few seconds at a repetition rate of 1 kHz. The asymmetrical ATI spectra driven by IR laser is observed with and without XUV pulses.

### Additional resources

No additional resources.

## Footnotes

Supplemental information can be found online at https://doi.org/10.1016/j.isci.2025.111899.

## References

[1] Ren X., Wang Y., Chang Z., Welch J., Bernstein A., Downer M., Brown J., Gaarde M., Couairon A., Kolesik M., Polynkin P. (2019). In-line spectral interferometry in shortwave-infrared laser filaments in air. Phys. Rev. Lett..

[2] Wang Y., Greene C.H. (2021). Two-photon above-threshold ionization of helium. Phys. Rev. A..

[3] Boltaev G.S., Kim V.V., Iqbal M., Abbasi N.A., Yalishev V.S., Ganeev R.A., Alnaser A.S. (2020). Application of 150 kHz laser for high-order harmonic generation in different plasmas. Photonics.

[4] Elizer S., Eichmann U. (2014). Steering neutral atoms in strong laser fields. J. Phys. B.

[5] Olofsson E., Carlström S., Dahlström J. (2021). Frustrated tunneling dynamics in ultrashort laser pulses. J. Phys. B.

[6] Nubbemeyer T., Gorling K., Saenz A., Eichmann U., Sandner W. (2008). Strong-field tunneling without ionization. Phys. Rev. Lett..

[7] Liu M., Xu S., Hu S., Becker W., Quan W., Liu X., Chen J. (2021). Electron dynamics in laser-driven atoms near the continuum threshold. Optica.

[8] Li Y., Xu J., Yu B., Wang X. (2020). Frustrated double ionization of atoms in strong laser fields. Opt Express.

[9] Chen S., Chen J., Paulus G.G., Kang H. (2020). Strong-field frustrated double ionization of argon atoms. Phys. Rev. A..

[10] Larimian S., Erattupuzha S., Baltuška A., K-Zeiler M., Xie X.-H. (2020). Frustrated double ionization of argon atoms in strong laser fields. Phys. Rev. Res..

[11] Eichmann U., Nubbemeyer T., Rottke H., Sandner W. (2009). Acceleration of neutral atoms in strong short-pulse laser fields. Nature.

[12] Eichmann U., Saenz A., Eilzer S., Nubbemeyer T., Sandner W. (2013). Observing Rydberg atoms to survive intense laser fields. Phys. Rev. Lett..

[13] Dunning F.B., Mestayer J.J., Reinhold C.O., Yoshida S., Burgdörfer J. (2009). Engineering atomic Rydberg states with pulsed electric fields. J. Phys. B.

[14] Chini M., Wang X., Cheng Y., Wang H., Wu Y., Cunningham E., Li P.C., Heslar J., Telnov D.A., Chu S.I., Chang Z. (2014). Coherent phase-matched VUV generation by field-controlled bound states. Nat. Photon..

[15] Zhao M., Wang Y., Quan W., Lai X., Liu H., Lu J., Liu X. (2021). High-lying Rydberg atoms surviving intense laser fields. Phys. Rev. A..

[16] Ortmann L., Hofmann C., Ivanov I.A., Landsman A.S. (2021). Controlling quantum numbers and light emission of Rydberg states via the laser pulse duration. Phys. Rev. A..

[17] Kang H., Chen S., Chen J., Paulus G.G. (2021). Frustrated double ionization of atoms in circularly polarized laser fields. New J. Phys..

[18] Wang B., Li X., Fu P., Chen J., Liu J. (2006). Coulomb potential recapture effect in above-barrier ionization in laser pulses. Chin. Phys. Lett..

[19] Zimmermann H., Eichmann U. (2016). Atomic excitation and acceleration in strong laser fields. Phys. Scr..

[20] Zhao J., Liu J., Wang X., Zhao Z. (2024). Twin-capture Rydberg state excitation enhanced with few-cycle laser pulses. Chin. Phys. Lett..

[21] Volkova E.A., Popov A.M., Tikhonova O.V. (2011). Ionization and stabilization of atoms in a high-intensity, low-frequency laser field. J. Exp. Theor. Phys..

[22] Li Q., Tong X.M., Morishita T., Wei H., Lin C.D. (2014). Fine structures in the intensity dependence of excitation and ionization probabilities of hydrogen atoms in intense 800-nm laser pulses, 2014. Phys. Rev. A..

[23] Lv H., Zuo W., Zhao L., Xu H., Jin M., Ding D., Hu S., Chen J. (2016). Comparative study on atomic and molecular Rydberg-state excitation in strong infrared laser fields. Phys. Rev. A..

[24] Xu S., Liu M., Hu S., Shu Z., Quan W., Xiao Z., Zhou Y., Wei M., Zhao M., Sun R. (2020). Observation of a transition in the dynamics of strong-field atomic excitation. Phys. Rev. A..

[25] Milošević D., Paulus G., Bauer D., Becker W. (2006). Above-threshold ionization by few-cycle pulses. J. Phys. B.

[26] Wang L., Lu X., Teng H., Xi T., Chen S., He P., He X., Wei Z. (2016). Carrier-envelope phase-dependent electronic conductivity in an air filament driven by few-cycle laser pulses. Phys. Rev. A..

[27] Ayuso D., Ordonez A.F., Ivanov M., Smirnova O. (2021). Ultrafast optical rotation in chiral molecules with ultrashort and tightly focused beams. Optica.

[28] Nakajima T., Watanabe S. (2006). Phase-dependent excitation and ionization in the multiphoton ionization regime. Opt. Lett..

[29] Yun H., Mun J.H., Hwang S.I., Park S.B., Ivanov I.A., Nam C.H., Kim K.T. (2018). Coherent extreme-ultraviolet emission generated through frustrated tunnelling ionization. Nat. Photon..

[30] Liu J., Zhao J., Huang Y., Wang X., Zhao Z. (2020). Dynamics of Rydberg states and terahertz waves generated in strong few-cycle laser pulses. Phys. Rev. A..

[31] Kübel M., Wustelt P., Zhang Y., Skruszewicz S., Hoff D., Würzler D., Kang H., Zille D., Adolph D., Paulus G.G. (2021). High-order phase-dependent asymmetry in the above-threshold ionization plateau. Phys. Rev. Lett..

[32] Chetty D., Glover R.D., Tong X.M., deHarak B.A., Xu H., Haram N., Bartschat K., Palmer A.J., Luiten A.N., Light P.S. (2022). Carrier-envelope phase-dependent strong-field excitation. Phys. Rev. Lett..

[33] Zhong S., He X., Ye P., Zhan M., Teng H., Wei Z. (2013). Effects of driving laser jitter on the attosecond streaking measurement. Opt Express.

[34] Robinson J., Haworth C., Teng H., Smith R., Tisch J., Marangos J. (2006). The generation of intense, transform-limited laser pulses with tunable duration from 6 to 30 fs in a differentially pumped hollow fibre. Appl. Phys. B.

[35] Zhang W., Teng H., Yun C.X., Ye P., Zhan M.J., Zhong S.Y., He X.K., Wang L.F., Wei Z.Y. (2014). Long-term stabilization of carrier-envelope phase for few cycles Ti: sapphire laser amplifier. Chin. Phys. Lett..

[36] Zhan M.J., Ye P., Teng H., He X.K., Zhang W., Zhong S.Y., Wang L.F., Yun C.X., Wei Z.Y. (2013). Generation and measurement of isolated 160-attosecond XUV laser pulses at 82 eV. Chin. Phys. Lett..

[37] Kling M.F., Rauschenberger J., Verhoef A.J., Hasović E., Uphues T., Milošević D.B., Muller H.G., Vrakking M.J.J. (2008). Imaging of carrier-envelope phase effects in above-threshold ionization with intense few-cycle laser fields. New J. Phys..

[38] Rathje T., Johnson N., Möller M., Süßmann F., Adolph D., Kübel M., Kienberger R., Kling M., Paulus G., Sayler A. (2012). Review of attosecond resolved measurement and control via carrier–envelope phase tagging with above-threshold ionization. J. Phys. B.

[39] Zhou Y., Guo L., Quan W., Wei M., Zhao M., Xu S., Xiao Z., Sun R., Wang Y., Lai X. (2021). Carrier-envelope phase dependence of high-order above-threshold ionization by few-cycle laser pulses. Phys. B..

[40] Ye P., He X., Teng H., Zhan M., Zhong S., Zhang W., Wang L., Wei Z. (2014). Full quantum trajectories resolved high-order harmonic generation. Phys. Rev. Lett..

[41] Zhang Y., Zille D., Hoff D., Wustelt P., Würzler D., Möller M., Sayler A.M., Paulus G.G. (2020). Observing the importance of the phase-volume effect for few-cycle light-matter interactions. Phys. Rev. Lett..

[42] Venzke J., Reiff R., Xue Z., Jaroń-Becker A., Becker A. (2018). Angular momentum distribution in Rydberg states excited by a strong laser pulse. Phys. Rev. A..

[43] Milošević D.B., Becker W. (2016). Improved strong-field approximation and quantum-orbit theory: Application to ionization by a bicircular laser field. Phys. Rev. A..

[44] Amini K., Biegert J., Calegari F., Chacón A., Ciappina M.F., Dauphin A., Efimov D.K., Figueira de Morisson Faria C., Giergiel K., Gniewek P. (2019). Symphony on strong field approximation. Rep. Prog. Phys..

[45] Becker W., Goreslavski S., Milošević D., Paulus G. (2018). The plateau in above-threshold ionization: the keystone of rescattering physics. J. Phys. B.

[46] Becker W., Milošević D.B. (2022). Elliptic dichroism in strong-field ionization of atoms subjected to tailored laser fields. Phys. Chem. Chem. Phys..

[47] Habibović D., Becker W., Milošević D.B. (2022). High-order harmonic generation by two linearly polarized laser fields with an arbitrary angle between their polarization axes. Phys. Rev. A..

[48] Paulus G.G., Lindner F., Walther H., Baltuska A., Goulielmakis E., Lezius M., Krausz F. (2003). Measurement of the phase of few-cycle laser pulses. Phys. Rev. Lett..

[49] Zhou Y., Quan W., Zhao M., Wang Z., Wang M., Cheng S., Chen J., Liu X. (2022). Improved carrier-envelope phase determination method for few-cycle laser pulses using high-order above-threshold ionization. Photonics.

[50] Khurmi C., Wallace W.C., Sainadh U S., Ivanov I.A., Kheifets A.S., Tong X.M., Litvinyuk I.V., Sang R.T., Kielpinski D. (2017). Measuring laser carrier-envelope-phase effects in the noble gases with an atomic hydrogen calibration standard. Phys. Rev. A..

