# Supplementary material for: Resolved frustrated tunneling ionization (FTI) in asymmetrical fast oscillation of above-threshold ionization spectrum
Source: iScience. 2025 Jan 25;28(3):111899. doi: 10.1016/j.isci.2025.111899 (PMC11907450; doi:10.1016/j.isci.2025.111899)
Supplement: Document S1. Figures S1–S9 [file mmc1.pdf]

iScience, Volume *28*

Supplemental information

Resolved frustrated tunneling ionization (FTI) in asymmetrical fast oscillation of above-threshold ionization spectrum

Lifeng Wang, Hao Teng, Fei Li, Bingbing Wang, Xiaoxin Zhou, Peng He, and Zhiyi Wei

Supplemental Materials

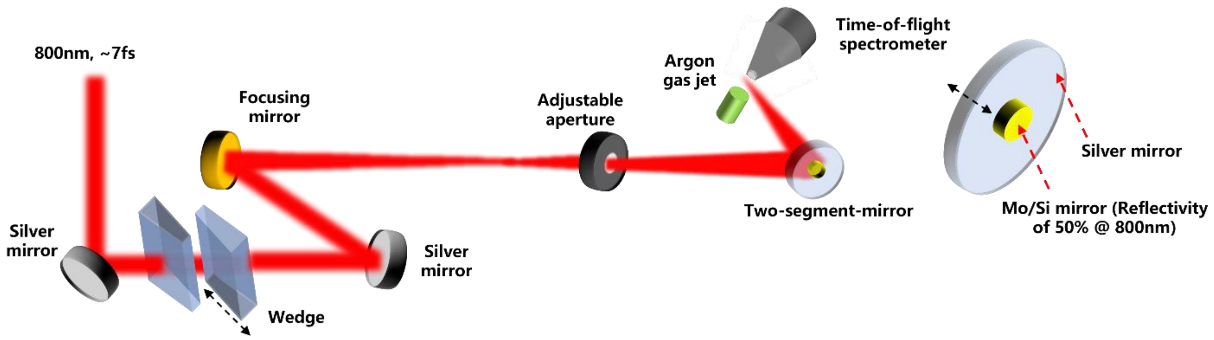

Fig. S1. Schematic of the experimental setup and the two-segment-mirror.

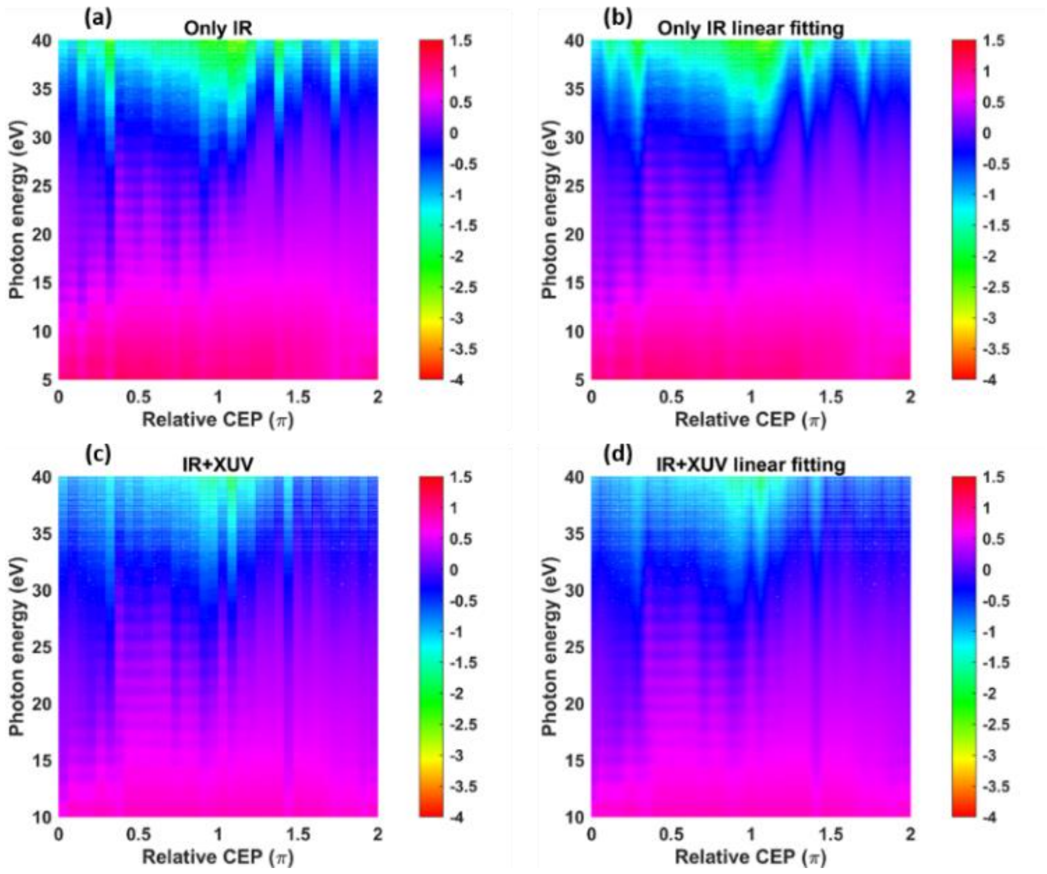

Fig. S2. Experimental data of fast oscillation in ATI spectra generated by IR and IR plus XUV. ATI spectrum driven by IR in log scale with original data (a) and linear fitting (b). ATI spectrum driven by IR and XUV in log scale with original data (a) and linear fitting (b).

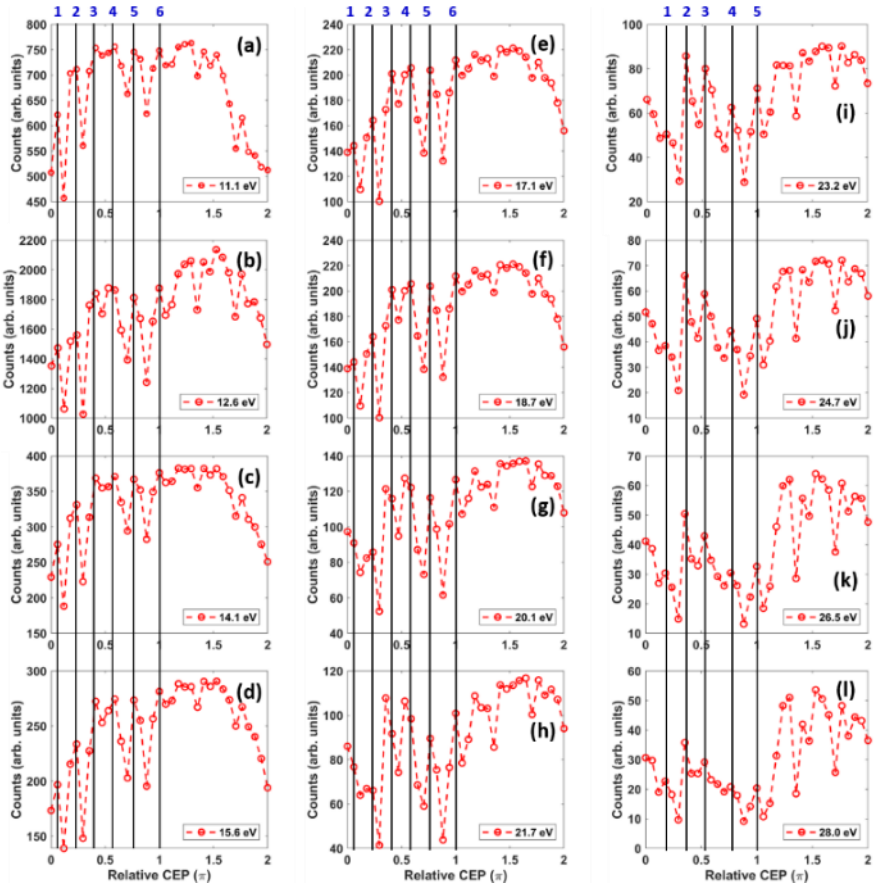

Fig. S3. ATI counts vs CEP in different electron energy range, 11.1 (a), 12.6 (b), 14.1 (c), 15.6 (d), 17.1 (e), 18.7 (f), 20.1 (g), 21.7 (h), 23.2 (i), 24.7 (j), 26.5 (k), 28.0 eV (l).

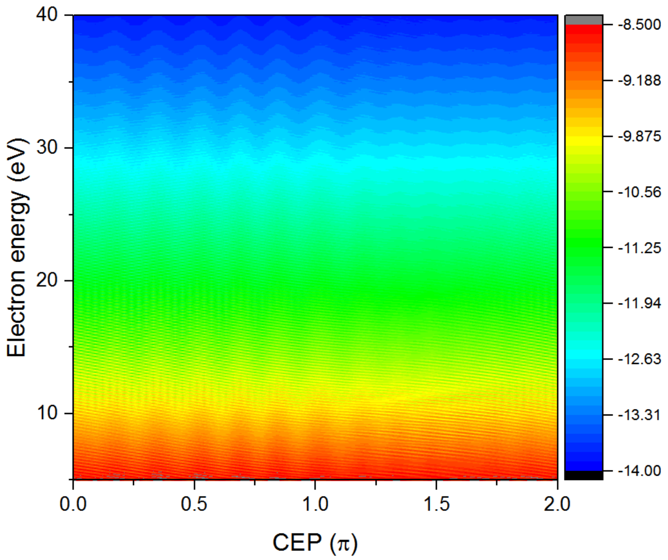

Fig. S4. Calculated ATI spectra vs CEP with focal volume averaging in step of 5.0×10^{12} W/cm^2.

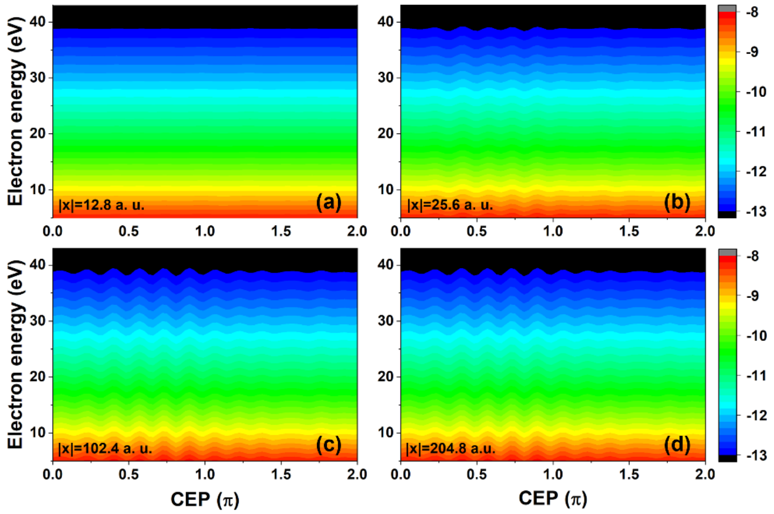

Fig. S5. Calculated ATI spectrum vs CEP for different absorbing boundaries in the frustrated tunneling ionization range

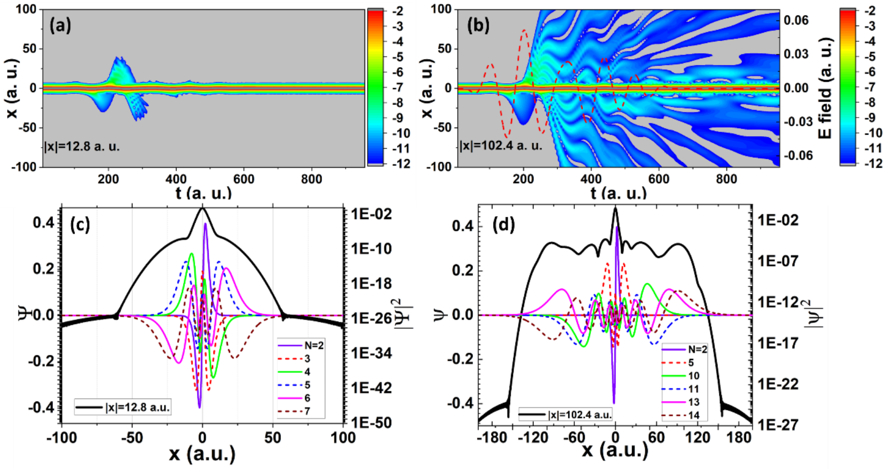

Fig. S6. Calculated electron wave packet evolution in space and time for boundaries of |x|=12.8 a. u. (a) and |x|=102.4 a. u. (b). Calculated final wavefunction and probability of excited states after laser pulse for 12.8 a. u. (c) and 102.4 a. u. (d).

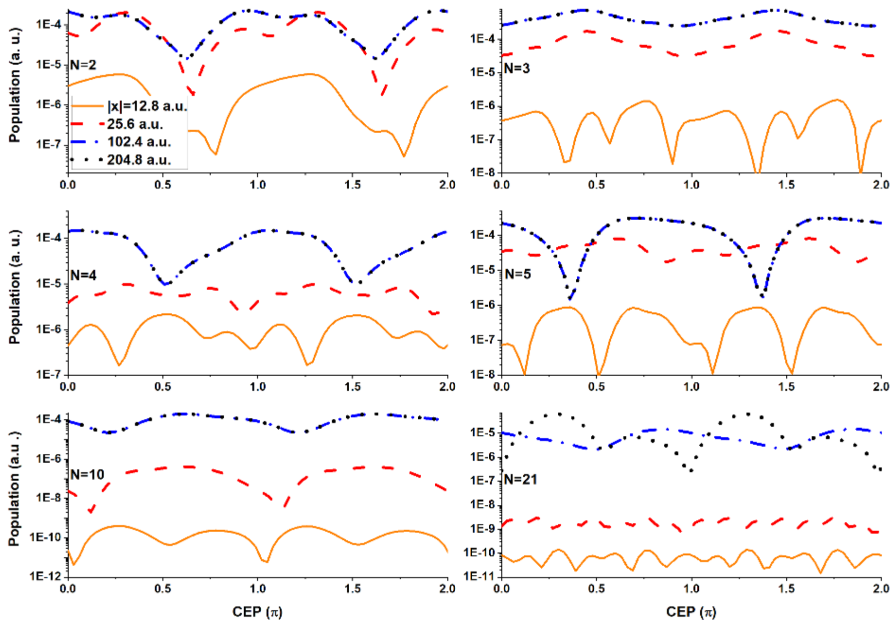

Fig. S7. Calculated population of excited states vs CEP for different absorbing boundaries, for N=2, 3, 4, 5, 10 and 21.

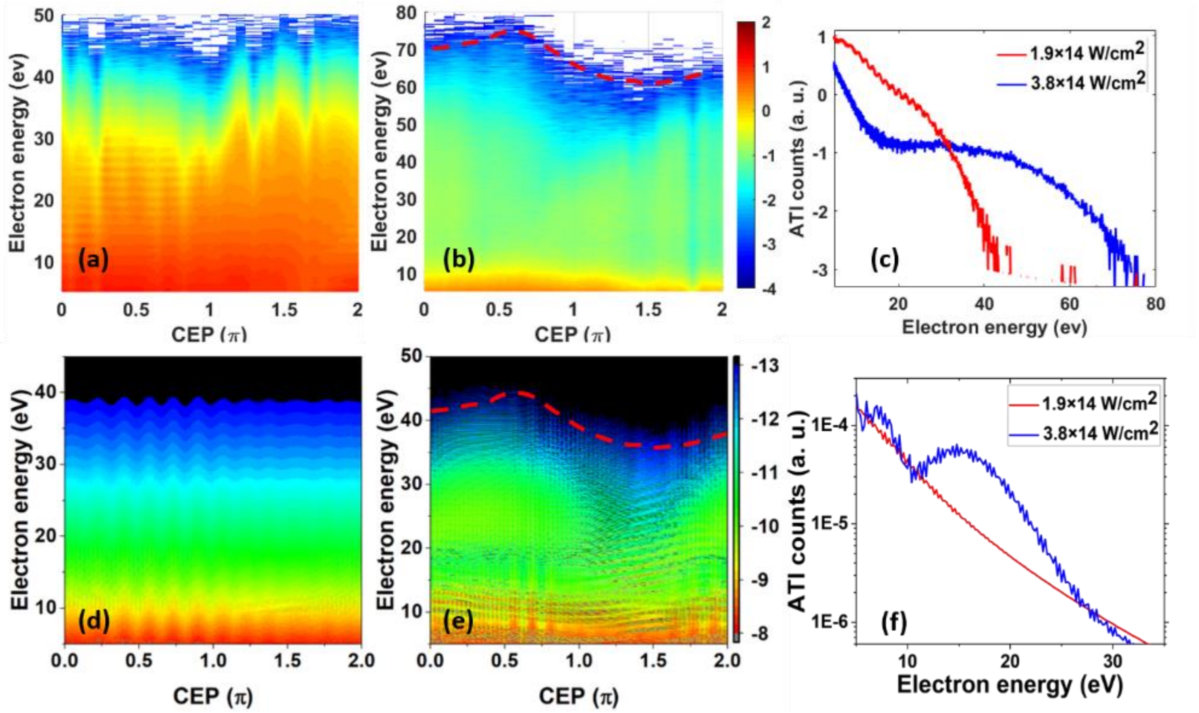

Fig. S8. Comparison of experimental data (a-c) with theoretical calculations (d-f) at two laser intensities.

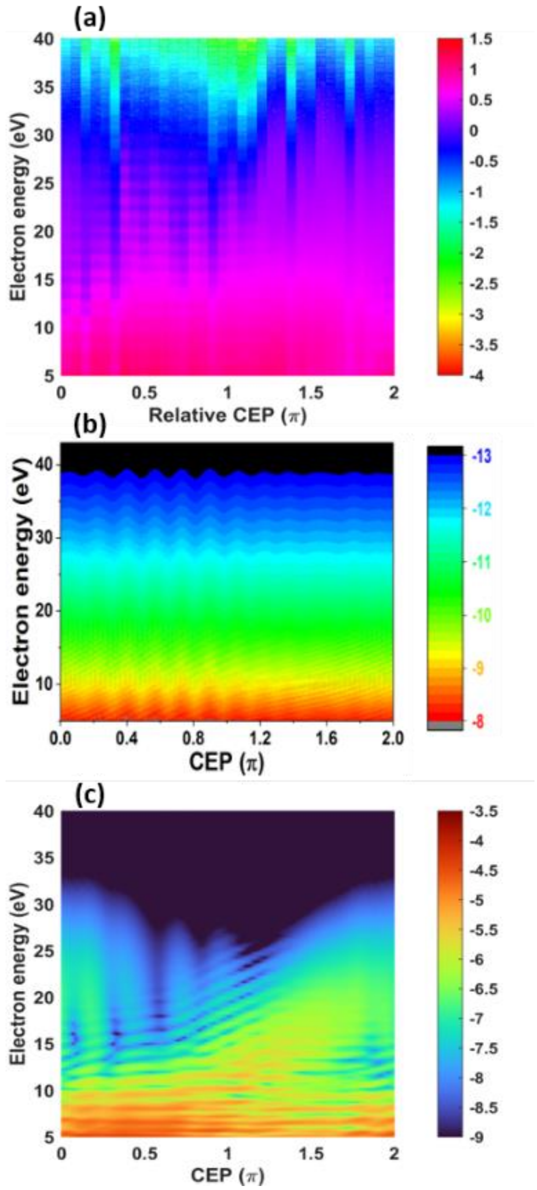

Fig. S9. Experimental and theoretical ATI spectra. The experimental ATI spectra vs CEP (a). Calculated results based on one-dimensional TDSE (b) and three-dimensional TDSE (c).

References:

[1] E. Goulielmakis, M. Schultze, M. Hofstetter, V. S. Yakovlev, J. Gagnon, M. Uiberacker, A. L. Aquila, E. M. Gullikson, D. T. Attwood, R. Kienberger, F. Krausz, and U. Kleineberg, Science. **320**, 1614 (2008).

[2] Y. Zhang, D. Zille, D. Hoff, P. Wustelt, D. Würzler, M. Möller, A. M. Sayler, and G. G. Paulus, Phys. Rev. Lett. **124**, 133202 (2020).

[3] Y. Zhou, L. Guo, W. Quan, M. Zheng Wei, M. Zhao, S. Xu, Z. Xiao, R. Sun, Y. Wang, X. Lai, J. Chen and X. Liu, J. Phys. B. **54**, 144008 (2021).

[4] S. Xu, M. Liu, S. Hu, Z. Shu, W. Quan , Z. Xiao, Y. Zhou, M. Wei, M. Zhao, R. Sun, Y. Wang, L. Hua, C. Gong, X. Lai, J. Chen, and X. Liu, Phys. Rev. A. **102**, 043104 (2020).
